# Psychiatric nurses advocating for the human rights of mental health care users in Gauteng

**DOI:** 10.4102/sajpsychiatry.v30i0.2233

**Published:** 2024-05-02

**Authors:** Nompumelelo Ntshingila, Annie Temane, Marie Poggenpoel, Masodi E. Makhale

**Affiliations:** 1 Department of Nursing, Faculty of Health Sciences, University of Johannesburg, Johannesburg, South Africa

**Keywords:** advocacy, human rights, mental health care users, psychiatric nurses, experiences

## Abstract

**Background:**

Psychiatric nurses play an important role in advocating for mental health care users such as advocating for the care, treatment and rehabilitation of mental health care users (MHCUs). Psychiatric nurses face various challenges while advocating for the human rights of MHCUs, particularly those unable to protect their rights because of the severity of their mental health conditions.

**Aim:**

This study aimed to explore and describe psychiatric nurses’ lived experiences in advocating for the human rights of MHCUs in the Gauteng province.

**Setting:**

The study was conducted within the primary healthcare (PHC) setting’s mental health services, Sedibeng District, Gauteng province.

**Methods:**

The study employed a qualitative, exploratory, descriptive and contextual research design. Three phenomenological focus group interviews were conducted, and audio recorded to collect data. Data were analysed using Tesch’s method.

**Results:**

Three themes emerged: (1) advocating for human rights was a strong push and an exhausting plea for psychiatric nurses in their attempts to voice and protect MHCUs’ rights; (2) MHCUs and mental health services were discriminated against and excluded by various stakeholders; mental health awareness should be raised and (3) training needs to be conducted as a matter of urgency in order to destigmatise mental illness from government to societal level.

**Conclusion:**

Psychiatric nurses experienced feelings of disempowerment, frustration and helplessness in advocating for MHCUs’ human rights.

**Contribution:**

The study’s findings will contribute to the body of knowledge in clinical psychiatric mental health practice on advocating for the human rights of MHCUs.

## Introduction

Mental illness, which is a debilitating condition, often renders mental health care users (MHCUs) incapable of their rights, necessitating advocacy from mental health care providers, particularly psychiatric nurses.^1,2^ This advocacy is rooted in the historical ill-treatment, stigmatisation and discrimination faced by MHCUs, dating back to 18th century in Britain and the United States during the custodial era.^3^ This mistreatment led to the establishment and introduction of laws such as Moral Management Treatment and advocacy movements in Europe and the United States.^4^ The emergence of advocacy movements on the African continent, including South Africa, became evident in the late 20th century.^5^

Globally, the human rights of MHCUs find recognition in the Universal Declaration of Human Rights.^6^ The incorporation of mental health into the United Nations’ Sustainable Development Goals and the World Health Organization’s Objective 3 signifies that MHCUs should be fairly treated^7,8,9^ and their rights should be respected. However, these individuals’ human rights are seldom advocated for despite these legislations. Notwithstanding these international frameworks, the advocacy for the human rights of these individuals is often insufficient, highlighting a gap between legislation and practical implementation.

A study in England that explored the experiences of psychiatric nurses in relation to human rights and ethical issues found that psychiatric nurses were powerless because of the lack of support, organisational limits, and ethical and moral conflicts.^10^ Another study that was conducted in European countries post coronavirus disease 2019 (COVID-19) pandemic found that nurses emphasised their importance in the role of patient advocacy, and although they experienced positive feelings in the form of professional pride and increased self-esteem, some of the nurses experienced emotional and ethical challenges because of advocating for the care of patients.^11^

Luca, Cavicchioli and Bianchi^12^ conducted a study in Switzerland and found that nurses experienced feelings of frustration, anger, powerlessness and discomfort because of the lack of multi-disciplinary team collaboration. These various feelings made it difficult to advocate for patients as members of the multi-disciplinary team, and the nurses were of different views regarding patient care.^12^ In Thailand, a study found that psychiatric nurses would identify problems such as unmet physical, social and financial needs of patients while they engaged in home visits; psychiatric nurses often feel it was an expectation for them to advocate for patients (‘being compassionate beyond the profession’), and this caused an increased workload and most nurses would experience burnout.^13^

Mental health care users globally and nationally are safeguarded by a range of laws.^14,15,16^ In South Africa, key protections include the *Mental Health Care Act,* No. 17 of 2002 (3)^14^ and the *Constitution of South Africa Act*, No. 108 of 1996, (2)^17^ affirming the entitlement of all human beings to basic human rights. The National Mental Health Policy Framework and Strategic Plan 2013–2020 of South Africa^18^ prioritise advocacy and human rights emphasising inter-sectoral collaboration. The *South African Nursing Act,* No. 33 of 2005 (2)^19^ mandates nurses to provide effective patient advocacy. The *National Health Act*, No. 61 of 2003^20^ promotes health care for all South Africans and protects the rights of individuals with disabilities, including MHCUs.

Psychiatric nurses in primary health care (PHC) settings face the challenge of advocating for the human rights of MHCUs.^15^ Despite returning to mental health services with persistent issues, such as family conflicts, housing struggles, medication unavailability, difficulties accessing social disability grants, work expulsions, and challenges in the right to treatment and treatment facilities, MHCUs often face obstacles in engaging in self-advocacy.^5,17,21^ The psychiatric nurses are advocating to the multi-disciplinary team, the government structures, and the community and family members. These difficulties are related to the human rights stipulated in the *Constitution of South Africa Act*, No. 108 of 1996 (2)^17^ and *Mental Health Care Act,* No. 17 of 2002 (3) of South Africa.^14^ The lack of advocacy for MHCUs’ human rights is evident in the Health Ombudsman of South Africa’s report following the deaths of over 100 MHCUs in unlicensed non-governmental organisations (NGOs).^22^

Dhai and McQuoid-Mason^23^ emphasise the importance of advocating for human rights within the legal framework, highlighting psychiatric nurses’ responsibility to protect MHCUs from harm resulting from the actions of others. This research addresses a significant gap by revealing a deficiency in advocacy for MHCUs’ human rights, particularly by mental health professionals, with specific reference to psychiatric nurses. The research was undertaken to gain an understanding of psychiatric nurses’ experiences in advocating for MHCUs’ human rights. This article therefore explored and described psychiatric nurses’ lived experiences in advocating for this population’s human rights.

## Research method and design

A qualitative, exploratory, descriptive and contextual design was used in the conduct of this research.^24^ The qualitative design facilitated the exploration of the phenomenon of interest in its natural setting, specifically within the mental health care service unit of a PHC setting. The qualitative method was used to gain an understanding of psychiatric nurses’ lived experiences in advocating for MHCUs’ human rights. Heidegger’s interpretive phenomenological approach was employed. This approach allowed the researcher to capture and interpret participants’ experiences,^25^ leading to the construction of themes. The contextual design ensured that the research was conducted within a specified context, as outlined in the study parameters.^26^

### Setting

The study was conducted at a PHC mental health services setting in the Sedibeng District of Gauteng province. The Sedibeng District is located in the South Gauteng province, covering an area of 4173 km^2^, bordered by three other provinces, namely the Free State, North West and Mpumalanga. There are 32 clinics in the region.^27^ The population at Sedibeng District sees an average of 1151 mental health care users per year.^28^

### Study population and sampling strategy

The study population consisted of psychiatric nurses who are working in mental health services and had experienced the phenomenon of interest, which was advocating for the human rights of MHCUs.^29^ The population comprised psychiatric nurses working in the secondary level of mental health services in a PHC setting in the Sedibeng District. These psychiatric nurses were in constant interaction with MHCUs. Sixteen psychiatric nurses were purposively sampled.^30^ The inclusion criteria were psychiatric nurses registered with the South African Nursing Council (SANC) under R425 of 1985 (as amended by R753 of 1988).^31^ Both females and males who were rendering mental health care services in a PHC setting were included in the study, and all participants had at least 2 years of experience working with MHCUs in Sedibeng District.

### Data collection

Data for the study were collected through phenomenological focus group interviews, complemented by observation and field notes to ensure data triangulation. Three focus groups, consisting of five members each for two groups and six members for one group, were interviewed separately. One focus group interview was conducted at the mental health services consulting room in a PHC setting, while other two focus group interviews were conducted in the training room of a Level 1 hospital after the multidisciplinary team meeting. The researcher opted for this approach to overcome challenges in meeting with the psychiatric nurses in their designated areas. The researcher had no relationship with the participants. The researcher was employed at a higher education institution and the participants were working in the PHC setting.

The interviews conducted for the study lasted between 45 and 90 min. Data were collected during the participants’ working time. Permission to collect data was obtained from the District office, and English language was used to collect data. Data were collected until saturation was reached when no new information was generated. The researcher provided an explanation of the purpose, and psychiatric nurses willingly signed consent forms, agreeing to have the sessions audio recorded to enhance trustworthiness, following the clarification of any questions. The participants were assured of their anonymity, confidentiality and privacy, and were informed of their right to voluntarily participate in the study. The researcher ensured to minimise harm to participants during the interviews. The researcher avoided bracketing her previous knowledge to gain a new understanding from the psychiatric nurses. Psychiatric nurses agreed to maintain anonymity and confidentiality during discussions, and participants were identified by focus group number (FG1), participant number (P) and gender (M/F) in the research report.

Therapeutic communication skills were used to help the researcher focus on the research question: ‘How is it for you to promote the human rights of psychiatric patients?’ The researcher also facilitated equal opportunity and participation among psychiatric nurses.

### Data analysis

In qualitative research, data collection and analysis occur concurrently. The data were analysed using Tesch’s eight steps of coding.^32^ The researcher made sense of the data by reading all transcriptions, field notes and observations. The researcher made notes from the information. The researcher grouped similar topics together to get the idea. Topics were abbreviated and arranged into codes next to relevant interviews, and the related topics were grouped in categories. A preliminary analysis was made by the researcher once each category was in one place and the meaning of themes was interpreted. The researcher met with the independent coder for a consensus discussion on the data analysis findings. The independent coder has a PhD and experience in qualitative research and data analysis. The researcher conducted a literature review to support the meaning of the themes and categories.^32^ Consensus between the researcher and co-coder was also reached before the supervisors confirmed the themes and categories. This methodological approach enhanced the depth and richness of the study findings.

### Rigour

The researcher adhered to trustworthiness criteria throughout the study, including credibility, transferability, dependability, confirmability and authenticity.^30^ Credibility was established through the active participation of psychiatric nurses in focus group sessions and interviews lasting for 45 min – 90 min. Triangulation was achieved by employing phenomenological focus group interviews, observations, field notes and audio recordings. Transferability was ensured through the purposive sampling of psychiatric nurses, and the inclusion of direct quotations to support findings. Dependability was maintained by providing a comprehensive description of the research methodology, and the analysis of psychiatric nurses’ lived experiences was grounded in existing literature. An independent co-coder was involved, and supervisors confirmed the coded data. Authenticity was maintained by keeping the transcripts, audio recordings, field notes and observations in a safe place. These would be used as evidence that the research was practically conducted.

### Ethical considerations

The study adhered to ethical principles, including autonomy, non-maleficence, beneficence and justice.^23^ Participants were safeguarded against harm during interviews and their confidentiality was strictly upheld. The participants voluntarily participated in the research after its purpose was explained to them. They signed consent forms for their participation in the focus group interviews and the use of an audio recorder, and their rights were respected. Research recordings are stored on the researchers’ laptop in a file which was password protected and are kept for 2 years after the study’s publication and will only be accessible to the researcher and supervisors. No compensation was offered to the participants.

Approval was obtained from the Research Ethics Committee (reference no.: REC-01-61-2017) and the Higher Degrees Committee at a university (HDC-01-40-2017) before the research commenced. Approval was also granted by the Gauteng Department of Health (GP-201710-037), the Director of Sedibeng District Health Services and the Mental Health Coordinator.

## Results

Sixteen psychiatric nurses participated in three phenomenological focus group interviews. Male and female participants, aged between 34 and 53, were included. Their experience in mental health ranged from 4 to 26 years. Three themes emerged from the analysed data, as presented in Table 1.^33^

**TABLE 1:** Themes and categories from analysed data.

Themes	Categories
Theme 1: Psychiatric nurses experienced advocacy to be a strong push and an exhausting plea in their attempts to voice and protect MHCUs’ rights and needs	Psychiatric nurses experienced advocating for MHCUs’ human rights in different ways Psychiatric nurses were advocating to different stakeholders Psychiatric nurses experienced affective responses to their advocacy attempts
Theme 2: Psychiatric nurses perceived MHCUs and mental health services were discriminated against and excluded by various stakeholders	Psychiatric nurses experienced that mental health services were excluded from physical and financial resources Psychiatric nurses experienced that MHCUs were excluded from human rights and supportive services Psychiatric nurses experienced that the exclusion of mental health services and MHCUs resulted in undesired outcomes
Theme 3: Psychiatric nurses recommended that mental health awareness should be raised and training needs to be conducted as a matter of urgency in order to destigmatise it from the government to the societal level	Psychiatric nurses recommended mental health awareness and training from the government to the societal level: ◦ Awareness and training for health care professionals ◦ Awareness and training for psychiatric nurses ◦ Awareness and training for MHCUs’ families ◦ Awareness and training for society Psychiatric nurses experienced being fulfilled when they rendered proper care to the MHCUs

*Source:* Makhale ME. Strategies to facilitate advocacy for human rights of mental health care users by psychiatric nurses [homepage on the Internet]. University of Johannesburg; 2020 [cited n.d.]. Available from: https://ujcontent.uj.ac.za/esploro/outputs/doctoral/Strategies-to-facilitate-advocacy-for-the/9913807 807691

MHCU, mental health care users.

### Theme 1: Psychiatric nurses experienced advocacy to be a strong push and an exhausting plea in their attempts to voice and protect mental health care users’ rights and needs

Advocating for the human rights of MHCUs has emerged as a formidable challenge for psychiatric nurses. The process demanded time, patience, teamwork and stakeholder involvement. However, the psychiatric nurses felt they were not supported in their attempts and were not achieving their goal of advocating for the MHCUs’ human rights.

#### Category 1.1: Psychiatric nurses experienced advocating for mental health care users’ human rights in different ways

Psychiatric nurses explained that many communication methods could be used to advocate for the human rights of MHCUs. These included accompaniments to speak on behalf of the MHCU, referral letters or memos, and telephonic discussions. One participant said: ‘One of the easiest way that I find is to write on the memo, unlike sending the patient on her own to other side’ (FG2, P3, F).

Another participant advocated for the human rights of MHCUs telephonically. ‘I took a phone, dialled police station’ (FG3, P3, F).

#### Category 1.2: Psychiatric nurses were advocating to different stakeholders

Psychiatric nurses said they had to advocate for MHCUs’ human rights to different stakeholders. These included government departments, emergency medical services, the police, PHC professionals, family members and society at large. One participant who communicated with emergency medical services said: ‘The EMS (Emergency Medical Services) claimed that the patients will break their equipment. How does the patient go to the hospital?’ (FG3, P1, F).

Another participant who communicated with PHC professionals explained:

‘I had to go and explain to them that this patient is medically ill. They have to attend to the medical problem of the patient irrespective of the fact that he is on Risperdal.’ (FG1, P3, F)

#### Category 1.3: Psychiatric nurses experienced affective responses to their advocacy attempts

Psychiatric nurses experienced feelings of disempowerment, frustration and helplessness in advocating for the human rights of MHCUs. Participants’ experiences with affective responses were congruent with the researcher’s observation and field notes during interviews, as one participant said: ‘We nurses are allowed to act up to so far. I wish we could be given more power to help the patients’ (FG2, P3, F).

Another participant, who experienced frustration in advocating for the human rights of MHCUs, said:

‘Well, promoting human rights of the patients is challenging, because you do it and you find others do not do it. It is frustrating because we don’t do it together.’ (FG2, P4, F)

One participant felt helpless when she referred a patient to the doctor, and the doctor did not attend to the stated problems. She explained: ‘The doctor just copied everything I wrote there and did the X-Ray and that was it, sent the patient home right away like that’ (FG1, P2, F).

### Theme 2: Psychiatric nurses perceived mental health care users and mental health services were discriminated against and excluded by various stakeholders

Mental health services are part of health services, as the patient is treated holistically. However, ignorance results in mental health services still being discriminated against and excluded by various stakeholders. The consultation rooms allocated to mental health services are not conducive for the treatment of MHCUs; there is an unequal distribution of funds from the national government to the services of mental health of users; the human rights of MHCUs were violated, and support services display a negative attitude towards the MHCUs. Psychiatric nurses faced difficulty in advocating for MHCUs’ human rights as they appeared mental health was not a priority, as discussed next.

#### Category 2.1: Psychiatric nurses experienced that mental health services were excluded from physical and financial resources

Physical and financial resources are needed in order to render effective mental health services so that MHCUs can receive quality care. Psychiatric nurses experienced an unequal distribution of these resources between mental health services and primary health services. This section discusses and examines these disparities.

**Exclusion from physical resources:** Psychiatric nurses expressed concern about the buildings and rooms that were allocated to mental health services. They stated that there were insufficient rooms and space. Moreover, some of the buildings and rooms promoted stigma against MHCUs as there was no privacy. Psychiatric nurses expressed the need for mental health facilities that will offer skills development, stimulation and rehabilitation for MHCUs. One participant explained the state of the mental health facility by saying:

‘Some of our facilities are not even friendly. Look at that other mental health clinic. The environment is not conductive at all. There is little or no privacy. The place is noisy, lots of disturbance and on and on.’ (FG2, P4, F)

**Exclusion from financial resources:** Financial resources are important to sustain all health services, including mental health services. Psychiatric nurses experienced that the health budget from the national government was unequally distributed, and mental health services received only a small portion. The following quotes reflect the stress psychiatric nurses experienced in terms of limited financial resources:

‘So most of us have good ideas, like taking the patients out during Easter. You can just take a *kombi*, take them to Rand Easter Show, to show that they are very important. They need social life also. But the government will say that they don’t have money for that. Our ideas are evaporating.’ (FG1, P1, M)

‘Mental health does not have budget. We are depending on PHC. If they are not happy, you don’t get.’ (FG2, P1, F)

#### Category 2.2: Psychiatric nurses experienced that mental health care users were excluded from human rights and supportive services

Mental health care users are human beings and are entitled to all the rights that every human being enjoys. This population is also entitled to all the supportive services available to other individuals. However, psychiatric nurses experienced that MHCUs were excluded from these services, as the discussion in this section indicates.

**Exclusion from human rights:** Psychiatric nurses experienced that MHCUs’ human rights were violated. One participant expressed her dissatisfaction with the treatment of MHCUs at an NGO:

‘The patients, they tell me, eat … you know when you peel your vegetables. That stuff. That’s what they cook for them. Or potatoes or pumpkins peels, everything you meant to put for your compost in order to make manure, that’s what they eat. I mean, the condition of that place!’ (FG1, P3, F)

The aforementioned statement indicates that MHCUs were deprived of their rights, as stipulated in the *Constitution of South Africa Act*, No. 108 of 1996 (2)^17^ and *Mental Health Care Act,* No. 17 of 2002 (3).^14^

**Exclusion from supportive services:** Psychiatric nurses experienced that supportive services like the police, emergency medical services (EMS) and other government services excluded mental health services. Participants explained:

‘I think the one is with EMS. They got this er … negative attitude towards mentally ill people, like, I’m not sure it’s the issue of stigma or lack of knowledge or lack of training.’ (FG1, P2, M)

‘I went to the housing department because *akere* [*you know*] the councillor did not want to help.’ (FG3, P2, F)

#### Category 2.3: Psychiatric nurses experienced that the exclusion of mental services and mental health care users resulted in undesired outcomes

Psychiatric nurses said MHCUs’ and mental health services’ exclusion from physical and financial resources, human rights and supportive services affected these individuals negatively. Participants also expressed that exclusion from the aforementioned services was affecting them, as they were unable to achieve their goal of advocating for the MHCUs’ human rights as mental health was not recognised as a priority. The following quotation emphasises psychiatric nurses’ experiences:

‘But when we look at our people, once they are diagnosed, and then the stigma is already there. This is one of challenges. I know one guy who has a profession in IT and he is a lecturer, but because he keeps on relapsing, at work they don’t want to hear anything about him anymore. He is economically excluded. He is just at home doing nothing to use those skills. So how do we go beyond that, go about helping them? You see.’ (FG1, P2, M)

### Theme 3: Psychiatric nurses recommended that mental health awareness should be raised and training needs to be conducted as a matter of urgency in order to destigmatise it from the government to the societal level

The psychiatric nurses recommended mental health awareness and training for the stakeholders as they were ignorant and lacked knowledge in the matter which made advocating for MHCUs challenging. Although the psychiatric nurses felt disempowered, they had a sense of satisfaction when advocating for the human rights of MHCUs.

#### Category 3.1: Psychiatric nurses recommended mental health awareness and training from the government to the societal level

As a result of their disempowerment, stakeholders’ ignorance and the Life Esidimeni challenges, psychiatric nurses expressed an urgent need to conduct mental health awareness and training sessions with stakeholders. A participant claimed:

‘The government should be given awareness about mental illness and mentally ill patients because they are not taken seriously, are of … hence the case of Life Esidimeni. It’s the government you know, the stigma and the discrimination is one that caused Life Esidimeni.’ (FG1, P4, F)

**Awareness and training for health care professionals:** ‘And I always think that these PHC sisters need some sort of mental training you know …’ (FG3, P2, F)

**Awareness and training for psychiatric nurses:** ‘Surely there are new developments in mental health, because when you send your staff for training you are also boosting their morale.’ (FG1, P3, F)

‘In some countries you go for even refresher courses, like how to advocate for better treatment for patients, our guys go for what we call retraining courses, management and risk assessment. So to empower people and go an extra mile will help to avoid these mishaps.’ (FG1, P2, M)

**Awareness and training for mental health care users’ families:** ‘Now you come across these difficult family members that will tell you that you favour the difficult patient! They tell us that he “is used to this, is undermining us.” It really takes time for families to understand.’ (FG2, P3, F)

‘Even family members, they need to be trained, they need to be skilled how to manage them in terms of resources. The patient is the only one with income, so his grant is shared among the family members.’ (FG1, P2, M)

**Awareness and training for society:** ‘… The other thing is the issue of NGOs that are not registered, you know. Mentally ill patients are dumped there. People are being abused.’ (FG1, P3, F)

#### Category 3.2: Psychiatric nurses experienced being fulfilled when they rendered proper care to the mental health care users

Although psychiatric nurses shared various experiences of disempowerment in advocating for the human rights of MHCUs, they also stated that they felt content in solving MHCUs’ problems:

‘Anyway beside all the problems, I sometimes feel happy and satisfied where I acted successfully to solve the patient’s problem. It’s like I won a trophy.’ (FG1, P3, F)

‘Truly speaking, advocating for my patients where I am working, was easy in terms of equipment.’ (FG2, P2, F)

## Discussion

This study aimed to explore and describe psychiatric nurses’ lived experiences in advocating for the human rights of MHCUs in the Gauteng province. A summary of the themes and categories that were identified from the analysis is presented in Table 1.^33^

The literature reveals a consistent approach among psychiatric nurses in advocating for MHCUs. Varghese^34^ emphasised the significance of speaking on behalf of MHCUs, portraying psychiatric nurses as the voice of the voiceless. The South African Depression and Anxiety Group also recommend additional strategies for communicating with people on behalf of MHCUs, namely using social media, telephone or one-on-one contact, brochures and newsletters.^35^ Advocacy for MHCUs’ human rights is necessary to enhance their lives and quality of health. However, advocating to stakeholders was a daunting task for psychiatric nurses because of their ignorance and the stigma attached to mental illness. The psychiatric nurses’ feelings of frustration and helplessness were consistent with the findings reported by Sobekwa and Arunachallam,^36^ who discovered that psychiatric nurses experienced negative feelings of being unappreciated and unsupported by stakeholders.

Participants in this study were concerned about insufficient space and the lack of privacy during therapeutic sessions with MHCUs. Participants also expressed concern over the lack of financial support from the national government for mental health services. The study by Lund, Kleintjies, Cooper, Petersen, Bhana, Fisher and MHaPP Research Programme Consortium^37^ determined that since the deinstitutionalisation of MHCUs, little has been done to improve mental health facilities in South Africa. As for the mental health budget, the World Health Organization Assessment Instrument for Mental Health Systems (WHO-AIMS)^38^ and Burns^3^ concur on the uneven budget distribution. Any attempt at excluding MHCUs’ human rights has a detrimental effect and is a violation of their rights.^30^ A report compiled by the Ombudsman stated that violations of this population’s human rights led to the abuse and deaths of MHCUs.^22^ Emsley and Seedat^39^ also discuss the implications of relapse because of the unavailability of medication.

The WHO-AIMS^38^ stated that the Department of Health has a coordinating body overseeing public education and mental health awareness campaigns. The government also uses NGOs in mental health education and awareness campaigns.^38^ However, despite its availability, increased awareness and training has yet to be implemented. It is recommended that health care professionals receive training in mental health, as there is a shortage of mental health care providers.^40,41^ Trained health care professionals will also integrate mental health into PHC, thereby reducing stigma and discrimination against mental illness.^42^

Raising mental health awareness and offering training for MHCUs’ family members is important as the family is the pillar of strength and support for this population.^43^ Family members will better understand the condition of their relatives who are diagnosed with a mental illness and will know how to help them. Training also helps to allay any negative feelings among family members themselves and assists them in accepting the MHCU’s condition. Mental health awareness and training for society reduce stigma and discrimination against MHCUs. The urgent need to train non-govermental organisations (NGOs) on mental health issues was identified to prevent a similar incident to that experienced by Life Esidimeni.^22^ Training the police force in mental health is also important as they are the first responders at the scene of disruptive MHCUs in the community.

Davoodvand, Abbaszadeh and Ahmad^44^ found that when advocacy is fulfilling, psychiatric nurses experience compassion and feelings of closeness to the MHCUs; they protect the human rights of MHCUs and prioritise these individuals’ mental health status. This statement is consistent with the psychiatric nurses’ utterances during the interviews in this study. When speaking about MHCUs, they felt unconditional love and presented non-judgemental remarks, acceptance and understanding of the MHCUs’ conditions.

### Strengths and limitations

This research excluded MHCUs’ and family members’ experiences of psychiatric nurses’ advocacy for their human rights. The psychiatric nurses who worked in the PHC setting were excluded from the study, as the focus was on those in the secondary level of mental health services.

### Recommendations

The findings of this study underscore the importance of integrating mental health into PHC to ensure a holistic approach to the treatment of MHCUs. Sufficient resources must be allocated to allow psychiatric nurses to render quality care to MHCUs. Training for all categories of nurses on mental health advocacy is also imperative and should be extended to family members and the community.

## Conclusion

This study revealed that psychiatric nurses encounter feelings of disempowerment, frustration and helplessness when advocating for the human rights of MHCUs. Because of psychiatric nurses’ affective experiences, strategies to improve their attempts at advocating for MHCUs’ human rights need to be developed. These strategies will empower psychiatric nurses with the necessary skills to advocate for the MHCUs’ human rights.
